# RELATIONSHIP BETWEEN PERSONALITY TRAITS AND PREFERENCE FOR COPING STYLES IN OLDER ADULTS

**DOI:** 10.1093/geroni/igae098.2503

**Published:** 2024-12-31

**Authors:** Momoho Kakuta, Yasuyuki Gondo, Takeshi Nakagawa, Yukie Masui, Kei Kamide, Kazunori Ikebe, Tatsuro Ishizaki, Yasumichi Arai

**Affiliations:** Osaka University, Suita, Osaka, Japan; Osaka University, Suita, Osaka, Japan; National Center for Geriatrics and Gerontology, Obu, Aichi, Japan; Tokyo Metropolitan Institute of Gerontology, Tokyo, Japan; Osaka University, Suita, Osaka, Japan; Osaka University Graduate School of Dentistry, Suita, Osaka, Japan; Tokyo Metropolitan Institute for Geriatrics and Gerontology, Tokyo, Japan; Keio University School of Medicine, Tokyo, Japan

## Abstract

Coping styles comprise various coping strategies roughly divided between primary and secondary control by active and passive attitudes. Personality traits are known to influence coping strategies. However, little is known about whether differential patterns of associations with personality traits emerge between primary and secondary control. This study aimed to clarify the relationship between personality traits and preference for coping styles in the octogenarian when the influence of personality would be maximized due to functional declines. Data were collected from 913 community-dwelling people (47.2% male) aged 78 to 82 years who participated in the Japanese longitudinal cohort study of Septuagenarians, Octogenarians, and Nonagenarians Investigation with Centenarians (SONIC). We analyzed the big five personality traits, SOC as the primary control, and positivity effect as the secondary control. We examined the correlation between personality traits and the two coping strategies and compared their independent correlation coefficients of the coping strategies for each personality trait. Only neuroticism was negatively correlated, and the others were positively correlated with both controls. There was no difference in the correlation coefficients of coping strategies on neuroticism and openness (n.s.), whereas extraversion (p =.000) and agreeableness (p =.048) had a significantly stronger correlation with the secondary control, and consciousness (p =.003) had a significantly stronger correlation with the primary control. The analysis indicated that the preference for coping styles differs by personality traits. We emphasized that higher neuroticism avoids using coping strategies, and higher consciousness and extraversion accelerate primary and secondary control, respectively.

